# Purple of Cassius

**Published:** 1853-04

**Authors:** 


### ARTICLE XI.

### *Purple of Cassius.*

Fine gold is to be dissolved in *aqua regia*, which consists of two parts of muriatic acid to one part of nitric acid, with a gentle heat. This is then the muriate of gold. I use six times the weight of acid to one of gold to dissolve it.

Pure tin is also to be dissolved in muriatic acid, as much as it will digest without heat.

To obtain the *purple of cassius*, drop thirty or forty drops of the muriate of gold into a bottle containing about half a pint of hot rain or river water, and immediately after about fifteen or twenty of the muriate of tin, and a purple powder will be precipitated, which is the article used to procure the life-like gum color.

In the manufacture of mineral teeth, I would recommend the use of a compound color for general use, both for body and enamel, as it will save much nicety in dividing the different materials into eighths and sixteenths of a grain, &c. The compound color I use gives a warm and life-like appearance to the teeth.

| | | |
|---|---|---|
| ℞ | Red oxide of titanium, . . | ℥ j. |
| | " " gold, . . . . . | ʒ ss. |
| | Oxide of platina, . . . | ʒ ss. |
| | Oxide of cobalt, . . . | gr. v. |
| | Uranium, Zircon, } . . . | a. a ℈ j. |
| | Black oxide of manganese, . | ℥ j. |
| | Nitre (pure crystals,) . . | ℥ ss. |

Grind all well together, and expose them to a white heat in a Hessian crucible for about one hour. In this state it should be taken from the fire and thrown into cold water with the crucible. When cold, break up and select the coloring matter from the fragments of the crucible, and grind into an impalpable powder. When a light yellow body is required, about five grains of white body to one of the compound color will suffice; or if a dark shade is wanted, add about one grain of muriate of ammonia, platina to five of compound color. In this way every desirable shade of color may be made.

Henry Villers.

The above directions may be of use to tooth manufacturers, but the "Delightful Kiln Drink," invented by Dr. Villers in 1827, before the temperance reformation had begun, and recommended by him, to prevent colds and fevers from exposure to the heat of the kiln, we think, in these temperance times, can be dispensed with.—*Dent. Recorder.*

